# Seroprevalence of Schmallenberg Virus Antibodies among Dairy Cattle, the Netherlands, Winter 2011–2012

**DOI:** 10.3201/eid1807.120323

**Published:** 2012-07

**Authors:** Armin R.W. Elbers, Willie L.A. Loeffen, Sjaak Quak, Els de Boer-Luijtze, Arco N. van der Spek, Ruth Bouwstra, Riks Maas, Marcel A.H. Spierenburg, Eric P. de Kluijver, Gerdien van Schaik, Wim H.M. van der Poel

**Affiliations:** Central Veterinary Institute, part of Wageningen UR, Lelystad, the Netherlands (A.R.W. Elbers, W.L.A.Loeffen, S. Quak, E. de Boer-Luijtze, R. Bouwstra, R. Maas, E. P. de Kluijver, W.H.M. van der Poel);; Netherlands Food and Consumer Product Safety Authority, Utrecht, the Netherlands (A.N. van der Spek, M.A.H. Spierenburg);; GD Animal Health Service, Deventer, the Netherlands (G. van Schaik)

**Keywords:** Orthobunyavirus, Schmallenberg virus, seroprevalence, within-herd prevalence, dairy cattle, the Netherlands, viruses, *Suggested citation for this article*: Elbers ARW, Loeffen WLA, Quak S, de Boer-Luijtze E, van der Spek AN, Bouwstra R, et al. Seroprevalence of Schmallenberg virus antibodies among dairy cattle, the Netherlands, winter 2011–2012. Emerg Infect Dis [serial on the internet]. 2012 Jul [*date cited*]. http://dx.doi.org/10.3201/eid1807.120323

## Abstract

Infections with Schmallenberg virus (SBV) are associated with congenital malformations in ruminants. Because reporting of suspected cases only could underestimate the true rate of infection, we conducted a seroprevalence study in the Netherlands to detect past exposure to SBV among dairy cattle. A total of 1,123 serum samples collected from cattle during November 2011–January 2012 were tested for antibodies against SBV by using a virus neutralization test; seroprevalence was 72.5%. Seroprevalence was significantly higher in the central-eastern part of the Netherlands than in the northern and southern regions (p<0.001). In addition, high (70%–100%) within-herd seroprevalence was observed in 2 SBV-infected dairy herds and 2 SBV-infected sheep herds. No significant differences were found in age-specific prevalence of antibodies against SBV, which is an indication that SBV is newly arrived in the country.

## Abstract 2

Seroprevalence was highest in the eastern part of the country, bordering Germany, where the virus was first identified.

During the last 2 weeks of August and the first 2 weeks of September 2011, dozens of veterinary practitioners in the Netherlands reported to a monitoring help desk (GD Veekijker) that several dairy herds with cows showed a sudden decrease in milk production, watery diarrhea, and occasional fever (*1*). The affected animals recovered, and extensive bacteriologic, virologic, and parasitologic testing of feces and blood samples of sick cows did not reveal an infectious cause for the clinical signs. Similar problems were reported at about the same time in Germany, and on November 18, 2011, the Friedrich Loeffler Institute (Greifswald, Germany) reported the detection of a novel orthobunyavirus that could be the cause of the clinical problems (*2*). Real-time reverse transcription PCR (RT-PCR), made available by the Friedrich Loeffler Institute, was used to test stored blood samples (N = 50) from the clinically diseased cattle in the Netherlands; 36% had positive test results. Since then, the virus has also been associated with congenital malformations in young animals (lambs, goat kids, and calves) (*3*).

The new virus is provisionally called Schmallenberg virus (SBV), or Shamonda-like virus. It is a RNA virus and shows 97% identity with Shamonda virus (SHAV) (small gene segment), 71% identity with Aino virus (medium gene segment), and 69% identity with Akabane virus (AKAV) (large gene segment) (*4*). All these viruses are part of the Simbu serogroup of the family *Bunyaviridae*, genus *Orthobunyavirus*. The Simbu serogroup is composed of several arthropod-borne viruses (arboviruses, including SHAV, AKAV, and Aino virus) transmitted by *Culicoides* spp. biting midges and mosquitos. SHAV was initially isolated from cattle and *Culicoides* spp. biting midges in Nigeria in the 1960s (*5**,**6*). In 2002, SHAV emerged in Japan and was isolated from *Culicoides* spp. biting midges and sentinel cattle (*7*). Japan has been considered an area to which several Simbu group viruses have been endemic in cattle since the 1970s (*8*).

Knowledge specifically related to SBV is limited; according to a risk assessment by the European Centre for Disease Prevention (http://ecdc.europa.eu/en/publications/Publications/231112_TER_Risk_assessement_Schmallenberg_virus.pdf), transmission of SBV to humans is considered unlikely but cannot be ruled out. Recently, serosurveys were conducted to assess zoonotic transmission of SBV in farmers and veterinarians in Germany and the Netherlands, who are likely to come in contact with the virus, but no infection was found (*9**,**10*).

In the Netherlands, reporting of suspected cases of SBV infection in animals (occurrence of arthrogryposis hydranencephaly syndrome in calves, lambs, and goat kids) is obligatory; a report is followed by confirmatory testing of brain tissue samples by RT-PCR. However, the observed suspected cases are likely an underestimation of the true rate of infection; in addition, infected livestock may give birth to healthy young animals, adding to the underestimation of the true rate of infection. Therefore, serodiagnostic studies are needed to detect past exposure to SBV in ruminant populations in the affected countries. Within weeks after the start of the SBV epidemic, a virus neutralization test (VNT) was developed at the Central Veterinary Institute (CVI). This test made it possible to quickly execute a seroprevalence study of antibodies against SBV in dairy cattle in the Netherlands.

## Materials and Methods

### Seroprevalence Sampling Design

To estimate the seroprevalence of SBV in the dairy cattle population in the Netherlands with considerable precision, we used the following preconditions for sample size calculation (*11*): an a priori expected prevalence of 50% (this will yield the highest possible sample size), a maximum allowable error in the prevalence estimate of ≈3%, a 95% confidence in the estimate, and a population size of ≈1.5 million head of dairy cattle (on the basis of 2012 census data of Statistics Netherlands, The Hague, the Netherlands). These conditions yielded a calculated sample size of >1,100 randomly selected dairy cattle.

Because dairy cattle and the premises on which they are housed are not distributed homogenously in the Netherlands, a stratified random sampling design with the 12 provinces in the Netherlands as a stratification level was set up to provide a representative sample. On the basis of census data of Statistics Netherlands (*12*), the stratified distribution of dairy cattle by province was used for setting up the sampling frame. The sampling frame comprised dairy cattle from which serum samples were collected during November 2011–January 2012 for monitoring testing of antibodies against bluetongue virus or as part of a specific surveillance investigation of 125 dairy farms to exclude introduction of notifiable animal diseases because of purchase of possibly contaminated bedding material from a third country. Serum samples from these dairy cattle were stored at CVI and were available for our study. The dairy cattle in our final sampling list (most drawn from the bluetongue monitoring set, completed with 37 randomly selected samples originating from 12 cattle herds from the surveillance investigation set) were randomly selected within each stratum (province) of the sampling frame proportional to the number of dairy cattle in each province. This process provided an accurate representation of dairy cattle of the target population in our sample.

Animals within a herd share common characteristics such as nutrition, housing, and exposure to disease pathogens (*13*). In the case of infectious diseases, common exposure to disease pathogens probably results in a common serologic status within herds. As a consequence, differences in prevalence between herds are larger than differences between animals within herds. Therefore, it is essential to sample relatively more herds and fewer animals within a herd than in a situation without clustering of disease events, as was shown for estimating the population prevalence for pseudorabies virus infection (*14*). A measure for agreement in serologic status between animals within a herd is given by the intraclass correlation coefficient µ (*15*). Because we presumed a high intraclass correlation with respect to serologic status of animals within herds (based on preliminary test results from a few infected herds), on average 2 dairy cattle (minimum 1, maximum 4) from the same dairy herd were included in the sampling list to prevent occurrence of too many cattle from the same herd. This selection procedure resulted in 1,123 samples from dairy cattle from 489 dairy herds.

**Figure 1:**
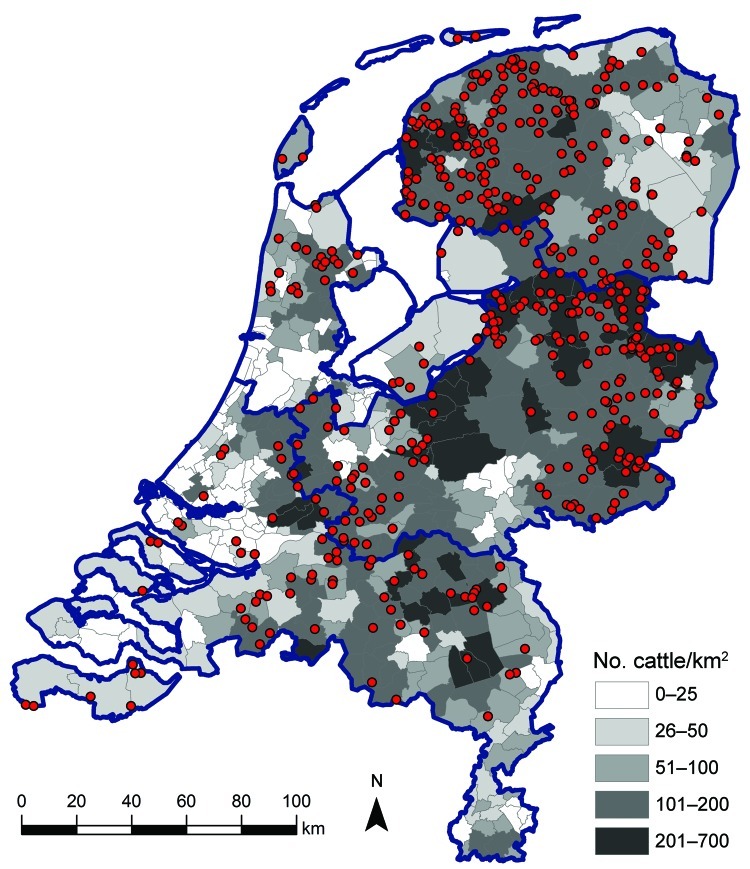
Geographic distribution of dairy herds from which 1–4 animals were sampled (red dots) in study of Schmallenberg virus seroprevalence, the Netherlands, 2011–2012. Cattle density is indicated by gray shading; blue outlines denote regional borders.

In addition to estimating seroprevalence of SBV in the dairy cattle population in the Netherlands, we estimated the seroprevalence in the dairy cattle population by 3 regions in the Netherlands to determine possible regional differences in seroprevalence. These regions were the northern part of the Netherlands (465 samples), comprising Groningen, Friesland, Drenthe, and Noord-Holland Provinces; the southern part of the Netherlands (196 samples), comprising Zeeland, Zuid-Holland, Noord-Brabant, and Limburg Provinces; and the central-eastern part of the Netherlands (462 samples), comprising Overijssel, Gelderland, Flevoland, and Utrecht Provinces. A cattle density map on municipality level was created on the basis of the number of cattle per municipality as received from the “Dienst Regelingen” from the Ministry of Economic Affairs, Agriculture and Innovation. Figure 1 shows the geographic distribution of dairy herds from which we tested, on average, 2 dairy cattle. The data indicate that our sampling was indeed representative for the geographic distribution of cattle in the Netherlands.

The mean age of cattle tested was 23 months (range 12–79 months); 60% were 20–24 months of age. To test possible differences in age-specific antibody prevalence for cattle in the northern, central-eastern, and southern regions, we defined 3 age cohorts: <18 months, 18–24 months, and >24 months. Date of birth and date of blood sampling were available for 1,085 head of cattle, which enabled us to calculate the age of the cattle at the date of blood sampling.

### Within-herd Seroprevalence

To gain insight into the within-herd seroprevalence of infected herds (based on RT-PCR test results of malformed lambs and calves that had been born), we sampled 2 cattle herds and 2 sheep flocks for comparison. We used the following preconditions for sample size calculation: an a priori expected prevalence of ≥70%, a maximum allowable error in the prevalence estimate of ≈5%, and a 95% confidence in the estimate.

Sheep flock 1 consisted of 800 ewes >1 year of age, 120 ewes <1 year of age, and 14 rams. The flock was located in the eastern part of the Netherlands. From the beginning of the study through December 30, 2011, a total of 41 lambs were born; 15 lambs (37%) were malformed. Clinical signs observed in the malformed lambs were arthrogryposis, ankylosis, scoliosis, torticollis, kyfosis, and hydranencephaly. From this flock, 60 ewes that had already lambed were tested.

Sheep flock 2 consisted of 81 ewes >1 year of age and 1 male ram. The flock was located in the southern part of the Netherlands. From the beginning of the study through February 7, 2012, a total of 30 lambs were born (15 female and 15 male); 2 lambs (13%) were malformed (1 female and 1 male). Clinical signs observed in the malformed lambs were arthrogryposis, ankylosis, torticollis, ataxia, and neurologic signs. From this flock, 35 ewes that had already lambed were tested.

Dairy herd 1 consisted of 58 dairy cattle >2 years of age and 40 young stock <2 years of age. The flock was located in the southwestern part of the Netherlands. From the beginning of the study through February 8, 2012, 1 calf was born malformed. Clinical signs observed in the malformed calf were arthrogryposis and ankylosis. From this herd, 34 dairy cattle were tested.

Dairy herd 2 consisted of 40 dairy cattle >2 years of age and 20 young stock <2 years of age. The flock was located in the northern part of the Netherlands. From the beginning of the study through January 30, 2012, 2 malformed calves (twins) were born. Clinical signs observed in the malformed calves were scoliosis and hydranencephaly. From this herd, 34 dairy cattle were tested.

### Statistical Analysis

Exact 95% CIs for estimated seroprevalences were calculated according to Fleiss (*16*). Differences in mean seroprevalence of antibodies against SBV of dairy cattle populations between regions in the Netherlands were tested with the 2-sample proportion test (*17*). Differences in age-specific mean prevalence of antibodies against SBV of dairy cattle in the northern, southern, and central-eastern region were tested with the 2-sample proportion test (*17*).

An intraclass correlation coefficient µ was calculated to measure the agreement in serologic status between dairy cattle sampled within the same herd. The intraclass correlation coefficient (minimum 0, maximum 1) was estimated by using analysis of variance, with herd as independent variable and the serologic status of individual animals (seropositive or seronegative) as dependent variable (*15*).

### Serologic Test

Serum samples were tested in a VNT against SBV (W.L.A. Loeffen et al., unpub. data). A virus isolate from brain tissue of a lamb, fourth passage on Vero (African green monkey kidney) cells, was used in the test, which was performed in flat-bottomed, 96-well microtiter plates on VERO cells. The medium used for cells and dilutions was Dulbecco minimal essential medium + Glutamax (GIBCO Invitrogen, Carlsbad, CA, USA), contained with 3% fetal calf serum and 1% penicillin and streptomycin at final concentrations of 100 IU and 100 µg/mL, respectively, in the medium. Serum samples were heated for 30 min at 56°C before testing. Serum samples were diluted in the test plate, starting from 1:4, followed by 2-fold dilutions up to 1:512 in volumes of 50 µL. Subsequently, virus (500 median tissue culture infective dose per well) was added to each well, also in a volume of 50 µL. After preincubation at 37°C for 1–2 hours, 20,000 cells per well were added in a volume of 100 µL. Plates were incubated for 5 days at 37°C in 5% CO_2_.

After 5 days, the plates were emptied and stained with amido black. The titer was determined as the reciprocal of the dilution in which 25%–100% of the monolayer was still intact. Titers >8 were considered positive on the basis of a prior validation in which a specificity and sensitivity of >99% were estimated with this cutoff. Control samples (positives and negatives) were included in each run of the test. Virus used in each run was back titrated in 24 columns of 4 dilutions each.

## Results

The estimated seroprevalence of antibodies against SBV in dairy cattle, winter 2011–2012, for the Netherlands (N = 1,123) was 72.5% (95% CI 69.7%–75.1%). The agreement in serologic status between dairy cattle sampled within the same herd in our prevalence study, as measured by the intraclass correlation coefficient, was high, 0.73. This finding indicates that in any particular herd, a strong tendency exists that either most cattle in that herd will be seropositive or most will be seronegative.

We found no statistically significant (p>0.05) differences in age-specific mean prevalence of antibodies against SBV of cattle in the 3 regions (Table). In the southern and northern regions, we found a slight trend of increased seroprevalence from the younger to the older age cohorts; in the central-eastern region, this trend was absent.

**Table:** Age-specific mean prevalence of antibodies against Schmallenberg virus among cattle, the Netherlands, 2011–2012

Age range, mo	No. cattle (prevalence, %)			
	Northern region	Central-eastern region	Southern region	Total
<18	7 (42.9)	13 (76.9)	13 (46.2)	33 (57.6)
18–24	331 (65.0)	337 (82.8)	151 (60.9)	819 (71.6)
>24	103 (72.8)	98 (80.6)	32 (68.8)	233 (75.5)

**Figure 2:**
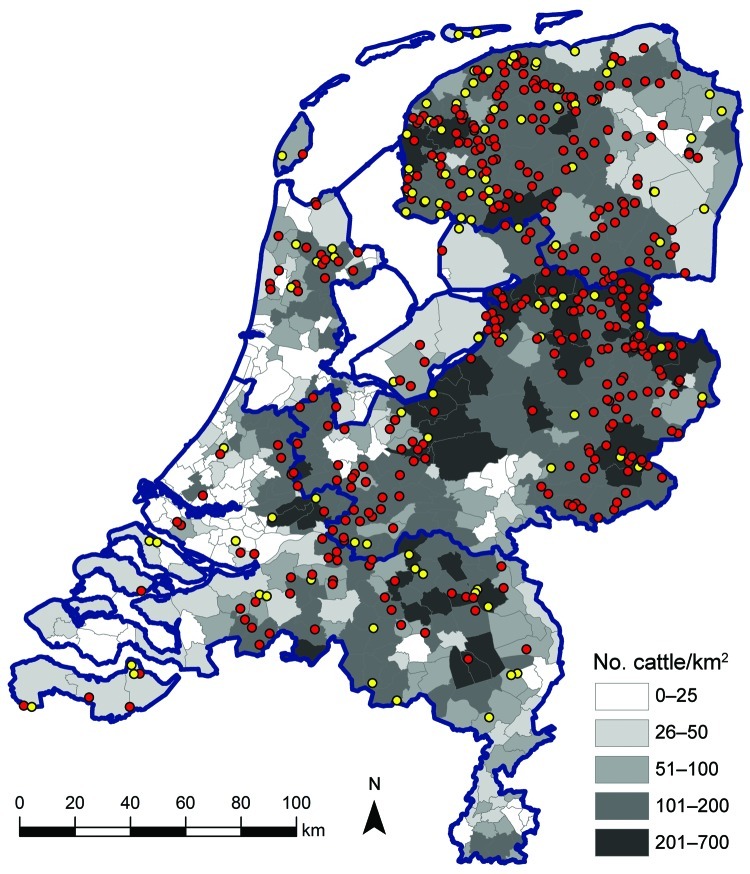
Geographic distribution of dairy herds sampled in study of Schmallenberg virus seroprevalence with positive results (>1 animals sampled tested seropositive; red dots) and negative results (all animals sampled tested seronegative; yellow dots), the Netherlands, 2011–2012. Cattle density is indicated by gray shading; blue outlines denote regional borders.

**Figure 3:**
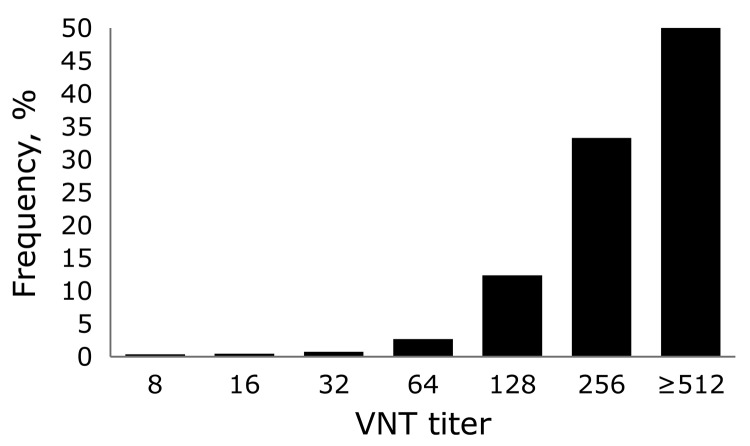
Frequency distribution of titers for serum samples (n = 814) positive for Schmallenberg virus antibodies by virus neutralization test (VNT) in study of Schmallenberg virus seroprevalence, the Netherlands, 2011–2012.

Figure 2 shows the geographic distribution of seropositive dairy herds (>1 cows sampled tested seropositive) and seronegative dairy herds (all cows sampled tested seronegative). These data indicate no association between cattle density and occurrence of seropositive or seronegative herds. Furthermore, the geographic distribution of seropositive and seronegative herds is random, showing no specific clusters of seropositive or seronegative herds. The estimated seroprevalence of antibodies against SBV in dairy cattle in the central-eastern part of the Netherlands (n = 462; seroprevalence 82.7%, 95% CI 78.8%–86.0%) was significantly (p<0.001) higher than the estimated seroprevalence of antibodies against SBV in dairy cattle in the northern (n = 465; seroprevalence 67.1%, 95% CI 62.6%–71.3%) and southern (n = 196; seroprevalence 61.2%, 95% CI 53.9%–68.0%) parts of the country. Figure 3 shows the distribution of VNT antibody titers against SBV of seropositive samples from dairy cattle; 50% of the samples showed a titer >512.

For testing of within-herd seroprevalence, in dairy herd 1, 25/34 cows tested seropositive (within-herd seroprevalence 73.5%, 95% CI 55%–87%); in dairy herd 2, all 34 cows tested seropositive (within-herd seroprevalence 100%, 95% CI 87%–100%). In sheep flock 1, 56/60 ewes tested seropositive (within-flock seroprevalence 93.3%, 95% CI 82%–98%); in sheep flock 2, 25/35 ewes tested seropositive (within-flock seroprevalence 71.4%, 95% CI 52%–85%).

## Discussion

We found a high seroprevalence of antibodies against SBV in dairy cattle in the Netherlands in the winter of 2011–2012, which indicates widespread exposure to SBV during the biting insect seasons of spring, summer, and fall 2011. Between the start of the investigation and last day of the surveillance period (March 29, 2012), a total of 782 calves with suspected SBV infection were tested by PCR in the Netherlands (Netherlands Food and Consumer Product Safety Authority, www.vwa.nl/onderwerpen/dierziekten/dossier/schmallenbergvirus); only 14% had positive test results. Combined with our study results on within-herd seroprevalence and the fact that a certain population of infected livestock would not produce malformed calves because the dams were infected outside the critical period of pregnancy, this finding illustrates the considerable underestimation of the true rate of infection in the population when only counting suspected cases.

The seroprevalence of antibodies against SBV in dairy cattle is significantly higher in the central-eastern part of the Netherlands than in the northern and southern parts of the country. This finding likely indicates that SBV was first introduced into the eastern part of the Netherlands and is supported by the fact that the first dairy herds reporting cows with clinical signs of SBV infection in September 2011 were located in the same areas (*1*).

We found no significant differences in age-specific mean prevalence of antibodies against SBV of cattle in the 3 regions, which indicates that SBV is newly arrived in the area. A clear increasing age-specific prevalence would have suggested that the virus had been there for 2–3 years but unrecognized earlier on. In the southern and northern region, there was a slight trend of increased seroprevalence from the young to the older age-cohorts, which can be expected because the young age cohort is housed inside for most of the time, preventing exposure to infected vectors. In the central-eastern region, this trend was absent, which is another indication that SBV was first introduced into the eastern part of the Netherlands.

Testing of serum samples banked during other studies before 2011 is planned to determine whether evidence exists of SBV infection before 2011. We could find no comparable seroprevalence studies on SBV or SHAV activity from other countries. However, a seroprevalence study conducted at the end of the New South Wales AKAV epidemic that occurred during April–October 1974 showed 80% seroprevalence in ≈4,000 serum samples from cattle (*18*). This finding illustrates that an outbreak season with another orthobunyavirus can result in a comparable level of infection to that found in our study.

Regarding SBV within-herd seroprevalence, our preliminary results indicate that, by the end of an outbreak season, most animals within an affected herd have been infected. Previous studies investigating AKAV outbreaks showed comparably high within-herd seroprevalence of antibodies in cattle in Australia: 77% in 1964 (*19*), up to 89% in 1971 (*20*), and 99% in 1988 in New South Wales (*21*). Furthermore, serologic investigations in the Kumamoto and Kagoshima Prefectures in Japan, where 98.3% of 119 tested cattle with neurologic signs were seropositive against AKAV, showed that 74.3% of cohabitated cattle without neurologic signs in these farms were also seropositive (*22*). Monthly sampling of sentinel cattle in Australia indicated that within 1–2 months after the start of sampling, 100% of the sentinel animals within herds were seropositive for AKAV exposure (*23**,**24*).

We cannot predict the progress of SBV during the coming months in the ruminant populations in the Netherlands. While a certain level of protection against new infection may be expected for naturally infected animals, but to our knowledge, no solid information on the protective capacity of SBV antibodies exists. In addition, in a population showing a seroprevalence of 70%, it should be assumed that a considerable portion of animals remain susceptible to SBV infection. Recent reports of SBV in *Culicoides* spp. biting midges from Belgium and Denmark implicated *C. obsoletus* complex and *C. dewulfi* midges as potential vectors in the transmission and spread of SBV (*25**,**26*). From experiences with other ruminant Simbu serogroup viruses in Asia and Australia, it may be assumed that, if previously uninfected animals are infected by vectors before mating, protection would be incurred against the occurrence of congenital malformations in newborns (*27*). Vaccination of the dams before they are mated would likely produce a similar protection; however, no vaccine for SBV is available.

Our study estimated seroprevalence at the population level and showed differences in seroprevalence among regions within the Netherlands. If an estimate of seroprevalence is desired at a more detailed regional level, a larger number of animals must be sampled and tested. This estimate will be feasible (cost- and labor-wise) when samples are tested with the VNT using a limited number of dilutions or an ELISA (less expensive and labor-intensive than the VNT) becomes available.

When designing our prevalence study, we assumed a high agreement in serologic status among dairy cattle sampled within the same herd. The observed within-herd prevalence and the high agreement in serologic status among dairy cattle sampled within the same herd in our study retrospectively indicate that sampling a relatively low number of animals within a herd and relatively more herds enables an accurate estimate of the overall seroprevalence of the dairy cattle population. These infection dynamic characteristics can be used by other research groups when designing future seroprevalence studies in the other SBV-affected countries.
